# The Nexus between Energy Consumption, Biodiversity, and Economic Growth in Lancang-Mekong Cooperation (LMC): Evidence from Cointegration and Granger Causality Tests

**DOI:** 10.3390/ijerph16183269

**Published:** 2019-09-05

**Authors:** Hongbo Liu, Shuanglu Liang

**Affiliations:** School of Economics, Yunnan University, Kunming 650091, China; hongboliu@ynu.edu.cn

**Keywords:** Granger causality tests, Lancang-Mekong Cooperation (LMC), energy consumption, sustainable development

## Abstract

This work is proposed to examine the relationships between energy consumption, biodiversity, and economic growth for China and five countries in the Indochina Peninsula, which are Cambodia, Laos, Myanmar, Thailand, and Vietnam, who also belong to Lancang-Mekong Cooperation, for the time span from 1991 to 2014. For this purpose, this work adopted autoregressive distributed lag (ARDL) through a dynamic simulation process. The estimation outcomes indicate that the nexus of the economic growth and energy consumption are diversified in fossil energy consumption and renewable consumption, respectively. The results are consistent with the fact that renewable energy is an alternative to fossil fuels, and traditional energy is still in the dominant position. This work is expected to serve as a first-hand examination on Lancang-Mekong Cooperation by adding innovative perspectives into existing research. Meanwhile, policy implications will also be discussed in this work.

## 1. Introduction

The past decades have witnessed a great increase in energy consumption [1]. The causal relationship between energy consumption and economic growth has been a popular topic among economic scholars all over the world. Not only are the developed countries implicated in energy consumption, but the emerging economies are pursuing economic development, which encourages surges of energy consumption. This work investigates China and five countries along the Lancang-Mekong River in order to extend the empirical research on the causal relationship between energy consumption and economic growth in these countries. For a long time in history, countries on the Indochina Peninsula were largely dependent on agriculture, but with economic development, their economic base has been broadly diversified, especially on tourism. Countries which belong to Lancang-Mekong Cooperation share common interests in not only water allocation but also environmental protection, due to their particular geographical location: They are all alongside the Lancang-Mekong River Basin [2], and therefore one country’s pollution emission could be easily transferred to others. Aside from this, economic interactions among these countries are becoming increasingly close, especially after the founding of the Belt and Road Initiative, in terms of international trade, foreign direct investment, as well as tourism communication [3]. All these could be epitomized by the Lancang-Mekong Cooperation mechanism; an innovative and developed initiative which is proposed to promote resource collaboration as well as economic communication [4].

The Lancang-Mekong Cooperation (hereafter LMC) was formed with the aim of contributing to the economic and sustainable development of its member countries. This cooperation is moving towards a new sub-regional cooperation structure, with a unique mechanism based on its constitutional strength and motivated by South–South cooperation. Considering the huge gap between China’s economic development and that of other LMC members [2,3,5], it is urgent for the promoter of this project to address the imbalanced conditions of its members, which also ignites this research. LMC is a multi-directional plan with aims including political and security issues, economic and sustainable development, social and culture cooperation, as well as supporting systems. Among these objectives, environmental protection is one of the key elements; LMC is proposed to promote the establishment of the Lancang-Mekong Environmental Cooperation Center, which is expected to synergize the environmental protection development plans of its member countries and formulate an LMC environmental protection strategy. Alongside this, LMC also emphasizes financial cooperation. It is proposed to work together to build a long-term, stable, sustainable, and diversified financial mechanism among sub-regional countries, and to encourage financial institutions to promote business operations, in order to contribute to regional economic communications and transnational investment [6].

LMC is not the only multilateral cooperative mechanism in this region; there existed quite a number of multilateral cooperation mechanisms in the Lancang-Mekong sub-region before the appearance of LMC [7]. For example, there is the US-Mekong Cooperation founded in 2009, advocated by Barack Obama in advancing the ‘Asia-Pacific Rebalancing’ strategy—the Mekong river was regarded as a significant strategic pivot of the US government. The Japan-Mekong Cooperation was also built in 2009 to promote “a new-type partnership for creating shared prosperity” [7]. Above all, there is the India-Mekong Cooperation, and the Republic of Korea (ROK)-Mekong Cooperation, effective among different countries in this region. These cooperation projects share both differences and similarities. Among these mechanisms, LMC as one of the newest founded ones, exposed high expectations among its member countries. Including China, members of LMC began to realize the importance of sustainable development during their cooperation.

In order to further the understand the cooperative mechanism among LMC member countries in terms of sustainable development, this research is proposed to serve as an initial study to understand the dynamic relationships between energy consumption and economic growth in LMC countries. This research is expected to provide suitable policy responses for these countries to face economic changes while keeping ecological friendliness and sustainable development.

There exists four hypothesizes in terms of explaining the Granger causal relationship between energy consumption and economic growth: Conservation hypothesis, growth hypothesis, feedback hypothesis, and neutrality hypothesis [8]. In detail, the ‘conservation hypothesis’ refers to a unidirectional Granger causality from economic growth to energy consumption; conversely, the ‘growth hypothesis’ suggests a unidirectional causality from energy consumption to economic development; and the ‘feedback hypothesis’ demonstrates that energy consumption and economic growth display bidirectional causality, going from energy consumption to economic growth, and vice-versa. Finally, ‘neutrality hypothesis’ is symbolized by the fact that there exists no causal relationship between the two variables of interest.

This paper contributes to a number of perspectives in recent literatures concerning energy consumption and economic growth in LMC countries. Firstly, unlike most of the former literature concerning LMC, whose focus were mostly on policy analysis or condition descriptions, this work made an innovation by constructing and reorganizing a cross-sectional database of six countries, which comprises economic development proxies, energy consumption, and biodiversity condition in LMC member countries. It facilitates the possibility of analyzing the complicated relationship between energy consumption and biodiversity in underlying countries. In detail, energy consumption in this research will be studied from two perspectives; the fossil fuel energy consumption, and the renewable energy consumption. As it is well known, China relies heavily on fossil fuels to keep its economy running and is one of the world’s largest CO_2_ emitters [9,10,11]. The Chinese government has realized this situation and is promoting the development of renewable energy consumption positively, which means China is expected to become one of the largest markets for renewable energy [12]. Therefore, it is both academically valuable and empirically necessary to study the nexus between energy consumption and economic growth in China, as well as its neighbor countries. Secondly, through the establishment of a reduced-form Granger causality relationship, this research does not rely on strong theoretical assumptions, which is different from the structure form of other studies. Thirdly, instead of using the EKC (Environmental Kuznets Curve) framework as many former researches did, this paper performed the Granger causality test to boost estimation robustness and model efficiency. Lastly, this present research further expands from a time series analysis to cross-sectional data research, which covers more information than most existing studies.

The rest of the paper is organized as follows: Section 2 presents a literature review in terms of four hypotheses concerned at explaining the Granger causality between energy consumption and economic growth. Section 3 examines the theoretical model, and the framework of econometric methodology. Section 4 contributes to data description and lays out the empirical models that are applied in the empirical analysis of this research. Section 4 examines the empirical results of the econometric analysis. Section 5 displays conclusions for this paper and their policy implications.

## 2. Literature Review

Following the fundamental research of Kraft and Kraft, researchers had been trying to extend the analysis on the relationship between energy consumption and economic growth, however, most of the studies had been subjected to continuous scrutiny [13]. During decades of development, studies of this area have witnessed transformations of various perspectives, including hypotheses, indicators, as well as methodologies. Given the large amount of previous studies in relevant disciplines, the literature review will be organized into three parts, in line with hypotheses, indicators adopted, and functions used to explain the theory.

### 2.1. Four Hypotheses

Among the large amount of former literature concerning energy consumption and economic growth, four possible causal relationships were detected: Conservation hypothesis, growth hypothesis, feedback hypothesis, and neutrality hypothesis [8]. Former research in this area demonstrates various outcomes in terms of different countries and through different methodologies, which can be observed in Table 1 and Table 2.

To conclude the two tables above, it is suggested that the adoption of different methodologies, empirical models, time periods, and variables could largely influence the causality results of each study. Not to mention that the causality results are varied among countries or regions [22,23]. That is to say, on the discipline of energy consumption and economic growth, they are not able to conclusively claim the causal directions, which means there is no homogeneous direction that can be predicted at either a global or single country level on whether energy consumption Granger causes economic growth or otherwise [24,25,26,27].

### 2.2. Proxy Variables

Two groups of variables are necessary in the empirical estimation of the Granger causality test in this research; energy consumption and economic growth. As for energy consumption, some scholars choose a single energy consumption variable, for example Raymond Li used coal consumption to analyze modern panel data on China and made a comparison among coastal, central and western regions in China’s energy consumption and economic growth [13]. Aside from this, electricity consumption is another popular index for relevant researches. For instance, Muhammad Shahbaz revisited the relationship between electricity consumption and economic growth in Pakistan, and came to a result that electricity conservation policies may unwittingly decline economic growth [28]. Unlike the above-mentioned cases that used single energy consumption data, Yamane Wolde-Rufael conducted his research through a more comprehensive dataset. His research found that there did exist a unidirectional Granger causal relationship running from coal, coke, and electricity to real GDP [29].

Another indispensable variable in related analyses is economic growth. James B. Ang [30] and Soytas [31] inaugurated the combined dynamic analysis between economic growth, energy consumption, as well as environmental pollution. These variables used to be indexed as to real GDP [32,33,34], GDP per capita [27], GDP of different industries [35,36], foreign direct investment, real fixed capital formation, total employment or labor force [36,37,38].

### 2.3. Methodology

In essence, most empirical research that is available on energy consumption and economic growth could be organized into a category named ‘reduced-form analysis’, in which the endogenous variables are described as a function of exogenous variables, usually without the structure or primitive policy invariant behavioral parameters [39,40]. Among the large number of researches, the cointegration test, the Johansen test for multivariate analysis, and the error correction model are frequently used [8,20,35,41,42].

To conclude, former studies concerning the relationship between energy consumption and economic growth have diversified conclusions due to various datasets from different countries or time spans, different models, and different variable selections. The inconclusiveness and variability of the conclusions of previous studies exposed the fact that, due to country characteristics, proxy variables, various datasets, different time ranges, as well as distinctive econometric methodologies, the outcomes of relevant researches could vary largely [23,43,44,45]. Despite the fact that mixing empirical results displays no unanimous conclusion on the existence or direction of Granger causality between energy consumption and economic growth, researches on the energy−growth discipline of certain countries or regions are still indispensable to provide the productive policy implications and recommendations for their sustainable development [46]. Therefore, this paper investigates the energy−growth nexus in the LMC countries by considering both renewable energy consumption and fossil energy consumption, biodiversity, and financial conditions of relevant countries. In this way, our research is proposed to contribute both to policymakers of underlying countries as well as to the literature [40,46].

## 3. Methodology and Data

The Granger causality test was proposed by C.W.J Granger and Robert Engel, aimed at testing the causal relationship between two variables using relatively short time spectrum through the introduction of panel data [47,48,49]. The above description also includes the major advantage of the methodology, which is enabling analysis while the data is not available in a long time series. As for its drawbacks, there is not much discussion in existing literature, but the usage of the particular method should be based on a complete beforehand test: Unit root test, cointegration test, and so on, which might be a burden for the beginners. The Granger causality test has been intensively utilized in examining the relationship between energy consumption and economic growth. Granger causality differs from the traditional ‘cause’ definition, which means Granger causality does not necessary imply ‘X causes Y’ [50]. In the Granger scope, it is more precisely to say that keeping all other conditions still, ‘X contains useful information for predicting Y with higher accuracy’ [51], or ‘X can be predicted more accurately through analyzing past values of Y instead of not using past values, while other conditions keeping constant’ [24,47,48].

### 3.1. Four Hypotheses

There exists four hypotheses in terms of explaining the Granger causality between energy consumption and economic growth: The conservation hypothesis, growth hypothesis, feedback hypothesis, and neutrality hypothesis [8]. In detail, the ‘conservation hypothesis’ refers to a unidirectional Granger causality from economic growth to energy consumption; conversely, the ‘growth hypothesis’ is suggested by a unidirectional causality from energy consumption to economic development; the ‘feedback hypothesis’ demonstrates that energy consumption and economic growth exposes a bidirectional causality relationship running from energy consumption to economic growth, and vice-versa. Finally, the ‘neutrality hypothesis’ is characterized by the fact that there exists no causal relation between the two variables we studied. In this research, it is proposed to test three hypotheses concerning the relationship between energy consumption and GDP, the relationship between energy consumption and financial development, and the relationship between energy consumption and biocapacity.

According to Engle R.F. and Granger C.W [47,48,49], for a linear combination of two or more non-stationary series with equivalent integration, it is possible to be stationary. With the existence of such a stationary linear combination, the series are regarded to be cointegrated, and long run equilibrium relationships stand [45,52].

$$\left[\begin{array}{c} {EC}_{t}\\ GDP_{t}\\ BIO_{t}\\ FI_{t} \end{array}\right]=\left[\begin{array}{c} \alpha_{1}\\ \alpha_{2}\\ \alpha_{3}\\ \alpha_{4} \end{array}\right]+\left[\begin{array}{cccc} A_{11,1} & A_{12,1} & A_{13,1} & A_{14,1}\\ A_{21,1} & A_{22,1} & A_{23,1}A_{12,1} & A_{24,1}\\ A_{31,1} & A_{32,1} & A_{33,1} & A_{34,1}\\ A_{41,1} & A_{42,1} & A_{43,1} & A_{44,1} \end{array}\right]*\left[\begin{array}{c} EC_{t-1}\\ GDP_{t-1}\\ BIO_{t-1}\\ FI_{t-1} \end{array}\right]+\ldots +\left[\begin{array}{cccc} A_{11,k} & A_{12,k} & A_{13,k} & A_{14,k}\\ A_{21,k} & A_{22,k} & A_{23,k} & A_{24,k}\\ A_{31,k} & A_{32,k} & A_{33,k} & A_{34,k}\\ A_{41,k} & A_{42,k} & A_{43,k} & A_{44,k} \end{array}\right]\;*\left[\begin{array}{c} EC_{t-k}\\ GDP_{t-k}\\ BIO_{t-k}\\ FI_{t-k} \end{array}\right]+\left[\begin{array}{cccc} A_{11,w} & A_{12,w} & A_{13,w} & A_{14,w}\\ A_{21,w} & A_{22,w} & A_{23,w} & A_{24,w}\\ A_{31,w} & A_{32,w} & A_{33,w} & A_{34,w}\\ A_{41,w} & A_{42,w} & A_{43,w} & A_{44,w} \end{array}\right]*\left[\begin{array}{c} EC_{t-w}\\ GDP_{t-w}\\ BIO_{t-w}\\ FI_{t-w} \end{array}\right]+\left[\begin{array}{c} \varepsilon_{1t}\\ \varepsilon_{2t}\\ \varepsilon_{3t}\\ \varepsilon_{4t} \end{array}\right]$$

where EC is energy consumption, GDP refers to annual percentage growth rate of GDP at market prices based on constant local currency, BIO is total biocapacity, and FI means financial condition, which will be represented by domestic credit provided by financial sector and broad money, respectively. $A_{i,j}$ are polynomials in the lag operator, ε_it_ is error-correction terms assumed to be uncorrelated and random with mean zero. Here are the hypotheses we are targeting:

$H_{01}$:$A_{12,1}=A_{12,2}=\ldots =A_{12,k}=0$, referring energy consumption does not Granger cause GDP growth.

$H_{02}$:$A_{21,1}=A_{21,2}=\ldots =A_{21,k}=0$, referring GDP growth does not Granger cause energy consumption.

$H_{03}$:$A_{13,1}=A_{13,2}=\ldots =A_{13,k}=0$, referring biodiversity does not Granger cause energy consumption.

The similar is true for other variables.

### 3.2. Data

**Figure 1:**
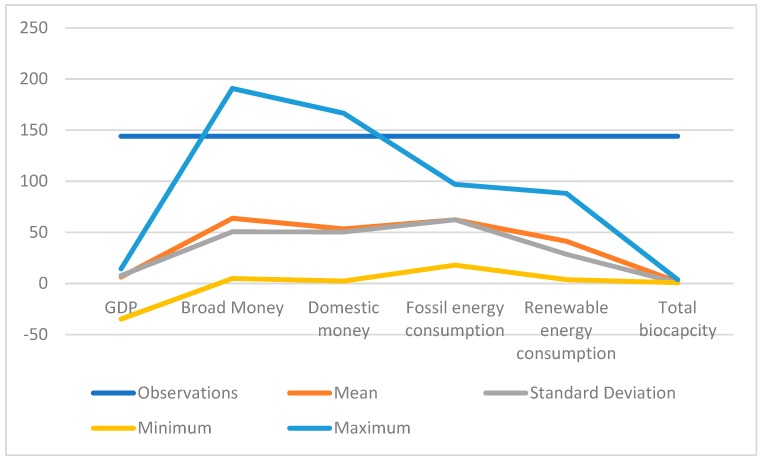
Data summary.

Time period ranges from 1991 to 2014, variables include life expectancy at birth, domestic credit provided by financial sector, broad money, GDP growth, energy use (kg of oil equivalent per capita), fossil fuel energy consumption (% of total final energy consumption), renewable energy consumption (% of total final energy consumption). Data came from World Bank. Biocapacity (GHA per person) data came from ecological footprint website. Data summary could be observed in Table 3 as well as in Figure 1.

## 4. Empirical Analysis

### 4.1. Unit Root Test

Estimating causality correlation using stationary data may lead to spurious outcomes [24,53]. In order to test the time series specialties of the parameters adopted in this analysis, it is proposed to conduct a nonstationary test including unit root test and cointegration test of the concerned variables [54,55]. Testing a unit root in energy consumption using all countries observations over the period concerned in this sample will be conducted through the augmented Dickey-Fuller (ADF) process [49]. As suggested by former studies of relevant disciplines, it is not recommended to include a trend factor in this particular analysis procedure. This is implied in STATA codes by adding no specification on ‘trend’ option, notwithstanding, ‘drift’ option was supplemented due to unique data acquisition. Likewise, it is proposed to use two lags in ADF regressions, which will dismiss cross-sectional specialty by using ‘demean’ option. In order to distinguish the time invariant property in cross-sectional data of the variables of interest, ADF is the most commonly used method, according to the literature review [56].

This study examines the stationarity of all dependent and independent variables by conducting an augmented Dickey-Fuller test (hereafter ADF) [56]. The ADF test is a popular and powerful method in time series analysis, which is commonly used in the literature. Nonetheless, with the improvement in time series-related studies, an upgraded method based on the original ADF test was designed by Elliot, Rothenberg, and Stock [57]. This innovative test is expected to be more powerful in terms of its explanatory ability and robustness than the traditional ADF test. The unit root tests based on panel data is expected to be more efficient than time series-base unit root test [58,59,60,61].

$$\Delta y_{t}=\alpha_{0}+\partial y_{t-1}+\sum_{i=1}^{P}\beta_{i}\Delta y_{t-1}+\varepsilon_{t}$$

where, *y_t_* is a vector for the time series variables in a particular regression, in our case, the variables under consideration, $\varepsilon_{t}$ is the error term, *P* refers to the optimal lag length.

The two hypotheses of unit root test in our specific research topics are as follows:

Ho: All panels contain unit roots.

Ha: At least one panel is stationary.

In Table 4, we can observe the results of the unit root test, taking into consideration the tests including: The Breitung test; Fisher-ADF test, which owns the null hypothesis that all the panels considered are stationary; as well as the Hadri test that all panels are stationary.

### 4.2. The Panel Cointegration Test

After the confirmation of the unit root, the next step is to implement the cointegration test. As a concept that existed for several decades, the earliest cointegration test invented was from Engle and Granger in 1987 [49,62]; Pesaran, Shin and Smith also provide a comprehensive autoregressive distributed lag (ARDL) test for integration. After that, there came the Johansen test, which is also a popular cointegration test to explore whether an equilibrium relationship exists among all parameters [38]. Johansen cointegration methodology enables an easier approach to estimate numbers of cointegration vectors and assists in determining the speed of coefficient adjustment.

In the second procedure, it is proposed to test a long-term relationship among underlying variables through panel cointegration mechanisms. In the current analysis, it is possible to perform the panel cointegration test with a new command ‘xtpedroni’ in STATA initiated by Timothy Neal [63].

In this study, we use the cointegration test claimed by Pedroni to testify the existence of a long-term correlation between biocapacity, energy consumption, financial condition, and GDP growth. The following unrestricted error correction models (UECM) are adopted for examination [64,65,66].

$$\begin{array}{c} \Delta lnEC=a+\overset{k}{\underset{i=1}{\sum }}\alpha_{i}\Delta lnEC_{t-i}+\overset{l}{\underset{i=1}{\sum }}\beta_{i}\Delta lnY_{t-i}+\overset{m}{\underset{i=1}{\sum }}\delta_{i}\Delta lnFI_{t-i}+\overset{N}{\underset{i=1}{\sum }}\gamma_{i}\Delta lnBIO_{t-i}\\ +\theta_{1}lnEC_{t-1}+\theta_{2}lnY_{t-1}+\theta_{3}lnFI_{t-1}+\theta_{4}lnBIO_{t-1}+\varepsilon_{t} \end{array}$$

$$\begin{array}{c} \Delta lnY=a+\overset{k}{\underset{i=1}{\sum }}\alpha_{i}\Delta lnEC_{t-i}+\overset{l}{\underset{i=1}{\sum }}\beta_{i}\Delta lnY_{t-i}+\overset{m}{\underset{i=1}{\sum }}\delta_{i}\Delta lnFI_{t-i}+\overset{N}{\underset{i=1}{\sum }}\gamma_{i}\Delta lnBIO_{t-i}+\theta_{1}lnEC_{t-1}\\ +\theta_{2}lnY_{t-1}+\theta_{3}lnFI_{t-1}+\theta_{4}lnBIO_{t-1}+\varepsilon_{t} \end{array}$$

$$\begin{array}{c} \Delta lnFI=a+\overset{k}{\underset{i=1}{\sum }}\alpha_{i}\Delta lnEC_{t-i}+\overset{l}{\underset{i=1}{\sum }}\beta_{i}\Delta lnY_{t-i}+\overset{m}{\underset{i=1}{\sum }}\delta_{i}\Delta lnFI_{t-i}+\overset{N}{\underset{i=1}{\sum }}\gamma_{i}\Delta lnBIO_{t-i}+\theta_{1}lnEC_{t-1}\\ +\theta_{2}lnY_{t-1}+\theta_{3}lnFI_{t-1}+\theta_{4}lnBIO_{t-1}+\varepsilon_{t} \end{array}$$

$$\begin{array}{c} \Delta lnBIO=a+\overset{k}{\underset{i=1}{\sum }}\alpha_{i}\Delta lnEC_{t-i}+\overset{l}{\underset{i=1}{\sum }}\beta_{i}\Delta lnY_{t-i}+\overset{m}{\underset{i=1}{\sum }}\delta_{i}\Delta lnFI_{t-i}+\overset{N}{\underset{i=1}{\sum }}\gamma_{i}\Delta lnBIO_{t-i}\\ +\theta_{1}lnEC_{t-1}+\theta_{2}lnY_{t-1}+\theta_{3}lnFI_{t-1}+\theta_{4}lnBIO_{t-1}+\varepsilon_{t} \end{array}$$

IIn the above equations, Δ refers to the first difference operator; ΔlnEC is the log of energy consumption, in our case, both fossile energy consumption and renewable energy consumption are considered; ΔlnY means the log of GDP growth; ΔlnFI is the log of financial condition, here represented by broad money and domestic money; and ΔlnBIO is the log of biodiversity. In order to detect whether a long-term relationship exists between the underlying variables, the F-test is adopted. The F-test is able to evaluable the significance of the lagged values of the parameters of interest [14]. Table 5 presents the panel cointegration test results, which includes panel v-statistic, panel rho-statistic, panel t-statistics, panel ADF-statistics, group rho-statistics, group v-statistics, and group ADF-statistics [67,68]. In this case, time dummies and trends are not included in the test process.

In Table 5, panel v-statistics represents non-parametric variance ratio statistics; panel rho-statistics indicates non-parametric test statistic analogous to the Phillips and Perron (PP) rho-statistic; panel t-statistics refers to the non-parametric statistic analogous to the PP t-statistic; and panel ADF-statistics means the parametric statistic analogous to the augmented Dickey-Fuller statistic [69].

### 4.3. IRF Test

The presence of a long-term correlation among energy consumption, GDP growth, financial condition, as well as biodiversity implies the existence of a Granger causality relationship at least in one direction [14,24,70].

Impulse response functions (IRFs) and forecast error variance decompositions (FEVDs) are two essential ambitions of vector autoregressive models. Both IRF and FEVD track the evolution of economic shocks through the calculation scheme. Nonetheless, in practical arithmetic calculations, researchers often suffer from the fact that the covariance matrix of the residuals in a VAR model is not diagonal, which indicates the coexisting correlation among errors. Therefore, the analysis of the transformation of one certain underlying variable is regarded to be inappropriate, because one transformation may take place concurrently with another innovation in the same system of interest [71].

### 4.4. Empirical Results

**Figure 2:**
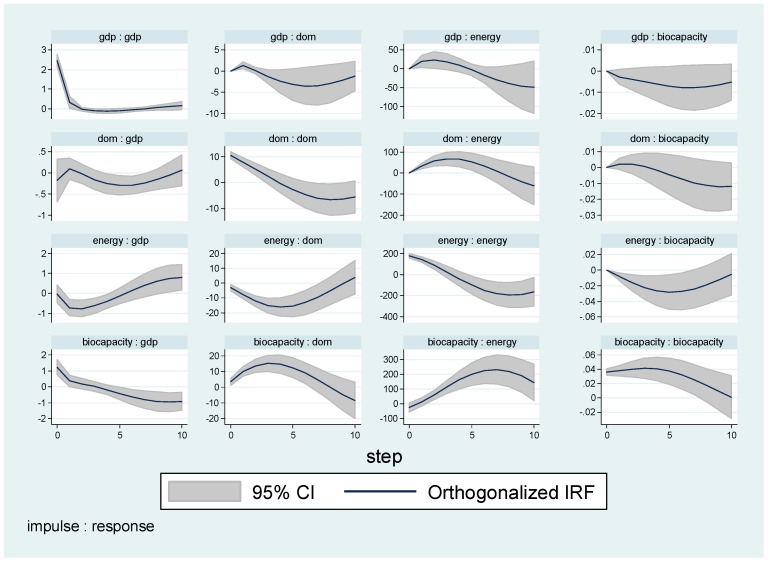
Impulse response function (IRF) test results of biocapacity, energy consumption, domestic money, and GDP.

**Figure A1:**
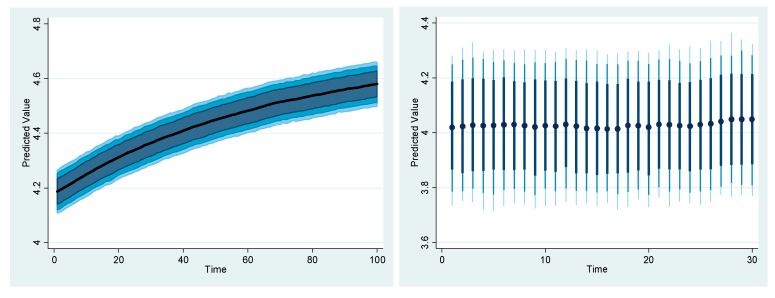
ln_fossileln_domln_biocapacity.

**Figure A2:**
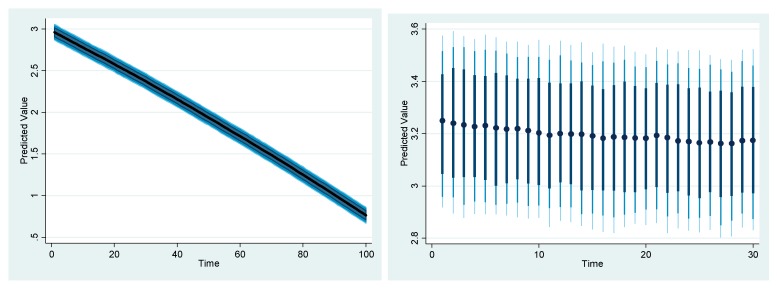
ln_renewln_domln_biocapacity.

**Figure A3:**
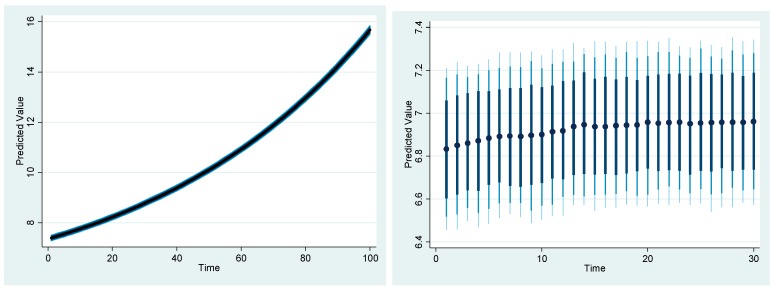
ln_energyln_domln_biocapacity.

Figure 2 displays the structural IRF of a shock in biocapacity, energy consumption, domestic money, and GDP on biocapacity, energy consumption, domestic money and GDP, respectively. It indicates that in this model a positive shock to biocapacity causes a slight increase, followed by a decrease and so on, until the effect dies out after roughly four periods. Although some of the impulse responses differ sharply, the response of GDP and energy consumption show similar domestic money shock across the two orderings. By contrast, biocapacity displays an inverse trend in structural IRF test. Besides, results of Figure A1, Figure A2 and Figure A3 displays the cointegration outcomes of fossil fuels consumption, renewable energy consumption and total energy consumption respectively.

Above all, this investigation confirms the fact that there is a strong and robust causal correlation from GDP to biocapacity, and vice versa. A similar conclusion is able to be made on domestic money and energy consumption, but a two-directional correlation could not be observed. However, comparison between renewable energy consumption and fossil energy consumption sheds some interesting outcomes, in general, energy consumption displays higher robustness and stronger correlations among variables. The relationship between energy use per capita and carbon emissions is significantly positive. The results we obtained through this model designation are in line with the well-accepted facts that renewable energy is a substitute to traditional fossil fuels and that traditional energy is still in the dominant position. On the other hand, the parameters of the renewable energy consumption proxy are insignificant, indicating that more attention should be paid to renewable energy, and its influence should be reinforced.

## 5. Discussion and Policy Implications

This work examined the relationship between energy consumption and economic growth for China and five countries in the Indochina Peninsula, which are Cambodia, Laos, Myanmar, Thailand, and Vietnam, in the time range from 1991 to 2014. For this purpose, this work adopted an autoregressive distributed lag (ARDL) through dynamic simulation process. This analysis is expected to serve as a first-hand examination on the Lancang-Mekong Cooperation by adding innovative perspectives into existing research.

For policy makers of a nation, understanding the relationship between energy consumption and economic growth, including renewable energy consumption, fossil fuel consumption, financial condition, as well as biodiversity, is extremely important. In the case of the LMC countries, given the region’s economic development condition, their energy consumption statement, as well as biodiversity situation, combining panel data analysis mechanisms are necessary. Empirically, the validity of the econometric model was tested through a panel dataset of six countries. In order to achieve this purpose, we performed panel unit root tests as well as a panel cointegration test. In addition, impulse response functions (IRFs) and forecast error variance decompositions (FEVDs), as two essential ambitions of vector autoregressive models, were adopted to empirically detect the correlations among underlying variables. Through this investigation process, it was possible to observe that there is a strong and robust causal correlation from GDP to biocapacity, and vice versa.

## 6. Conclusions

To conclude, research outcomes of this work highlight the importance of sustainable development through empirical analysis among LMC member countries for the first time. At the beginning, through testing the four hypotheses in LMC countries, it is proposed that the underlying countries should balance economic development, energy consumption, and biodiversity. Secondly, it is suggested that LMC countries should increase their consciousness in sustainable development, in other words, these countries are expected to build up more specific, more detailed, and more stringent policies to promote clean energy consumption. Lastly, most of LMC countries belong to the low- or middle-income nations, hence it is recommended that these countries reevaluate their industry constitution and place more emphasis on renewable resources in their production and consumption process.

It is recommended for further research to make modifications of the measurement of biocapacity, in addition, field investigation of this specific area is expected to shed inspiring results as these countries’ bioresources are relatively abundant. What is more, in line with the empirical results of most former literature, our research is unable to provide confirmative evidence of Granger causality [72]. More comprehensive comparisons between LMC member countries and those who are geographically connected but are not in the LMC group is proposed to demonstrate consequential results.

## Figures and Tables

**Table 1:** Single country case for Granger causality studies.

Author	Country	Time Period	Methodology	Conclusion
Hooi Hooi Lean, Russell Smyth [14]	Malaysia	1970–2008	ARDL (autoregressive distributed lag); TYDL (Toda and Yamanoto and Dolado and Lutkepohl)	Economic growth→Electricity generation
David Stern [15]	US	1945–1995	Multivariate cointegration	Energy→GDP
Khalifa H. Ghali [16]	Canada	1961–1997	Vector error-correction	Output growth→Energy use
Muhammad Shahbaz [17]	Pakistan	1972–2011	VECM Granger causality	Renewable energy consumption→Economic growth;nonrenewable energy consumption→Economic growth
Chi Zhang, Kaile Zhou [1]	China	1978–2016	Vector error correction model	Energy consumption→Economic growth;

Notes: In this frame, → represent unidirectional Granger causality.

**Table 2:** Country groups case for Granger causality studies.

Author	Country Group	Time Period	Methodology	Conclusion
Anis Omri, etc. [18]	MENA (Middle East and North Africa Countries) countries	1990–2011	Dynamic simultaneous equation	CO_2_ emission→GDP
Nicholas Apergis, James Payne [19]	EOCD countries	1985–2005	Panel Cointegration, error correction model	Renewable energy consumption→Economic growth
Angeliki N. Menegaki [20]	27 European countries	1997–2007	Random effect model for cointegration	Neutrality hypothesis
Can Tansel Tugcu, etc. [21]	G7 countries	1980–2009	Autoregressive distributed lag (ARDL)	No causal relationship

Notes: In this frame, → represent unidirectional Granger causality.

**Table 3:** Data summary.

Variable	Observations	Mean	Standard Deviation	Minimum	Maximum
GDP	144	6.35	7.70	−34.81	14.23
Broad money	144	63.73	50.59	4.89	190.75
Domestic money	144	53.38	50.35	2.37	166.50
Fossil energy consumption	144	62.35	62.35	17.92	96.95
Renewable energy consumption	144	41.26	28.57	3.82	88.11
Total biocapacity	144	1.56	0.68	0.72	3.74

**Table 4:** Results of unit root test.

Variables	Breitung	Fisher-ADF	Hadri
GDP growth	−1.61 (0.05)	24.26 (0.02)	12.91 (0.00)
Broad money	3.32 (0.99)	1.91 (0.99)	26.73 (0.00)
Domestic money	3.32 (0.99)	5.40 (0.94)	15.28 (0.00)
Fossil energy consumption	3.18 (0.99)	25.13 (0.01)	16.53 (0.00)
Renewable energy consumption	3.49 (0.99)	22.21 (0.04)	17.62 (0.00)
Total biocapacity	1.91 (0.97)	4.64 (0.97)	26.02 (0.00)

**Table 5:** Panel cointegration test results.

Observations: 144 Number of Panel Units: 6	
Test Name	Test Statistics
Panel v-statistics	0.06428
Panel rho-statistics	0.04571
Panel t-statistics	−0.5923
Panel ADF-statistics	0.1192
Group rho-statistics	0.689
Group v-statistics	−0.3259
Group ADF-statistics	0.615

Note: all test statistics are distributed N (0, 1), under a null of no cointegration, and diverge to negative infinity (save for panel v).

## Abbreviations

Alternative methodologies other than standard Granger causality tests: Engle-Granger (1987); Johansen-Juselius (JJ) (1988, 1990); Hansen-Seo (2002) threshold Cointegration; Pedroni (1999, 2004) Cointegration; Westerlund (2006) Cointegration; Pesaran and Shin (1999) and Pesaran et al. (2001) ARDLtest (ARDL); Toda-Yamamoto (1995) (TY), and Dolado and Lütkepohl (1996). Abbreviations for models: VAR = Vector autoregressive model and VEC = Vector error correction model.

## Appendix A

Note 1: Black dotted line shows average predicted value. Shaded area shows (from darkest to lightest) the 75, 90, and 95 percent confidence intervals.

Note 2: Dots show mean change in predicted value from sample mean. Shaded area shows (from darkest to lightest) the 75, 90, and 95 percent confidence intervals.
